# Mechanisms Involved in the Improvement of Lipotoxicity and Impaired Lipid Metabolism by Dietary α-Linolenic Acid Rich *Salvia* *hispanica* L (Salba) Seed in the Heart of Dyslipemic Insulin-Resistant Rats

**DOI:** 10.3390/jcm5020018

**Published:** 2016-01-28

**Authors:** Agustina Creus, María R. Ferreira, María E. Oliva, Yolanda B. Lombardo

**Affiliations:** Department of Biochemistry, School of Biochemistry, University of Litoral, Ciudad Universitaria, Paraje El Pozo, CC 242, (3000) Santa Fe, Argentina; agustinacreus@gmail.com (A.C.); mrferreira@fbcb.unl.edu.ar (M.R.F.); meoliva@fbcb.unl.edu.ar (M.E.O.)

**Keywords:** α-linolenic acid (ALA), cardiac muscle, lipotoxicity, dyslipidemia, insulin resistance, high-sucrose diet

## Abstract

This study explores the mechanisms underlying the altered lipid metabolism in the heart of dyslipemic insulin-resistant (IR) rats fed a sucrose-rich diet (SRD) and investigates if chia seeds (rich in α-linolenic acid 18:3, *n*-3 ALA) improve/reverse cardiac lipotoxicity. Wistar rats received an SRD-diet for three months. Half of the animals continued with the SRD up to month 6. The other half was fed an SRD in which the fat source, corn oil (CO), was replaced by chia seeds from month 3 to 6 (SRD+chia). A reference group consumed a control diet (CD) all the time. Triglyceride, long-chain acyl CoA (LC ACoA) and diacylglycerol (DAG) contents, pyruvate dehydrogenase complex (PDHc) and muscle-type carnitine palmitoyltransferase 1 (M-CPT1) activities and protein mass levels of M-CPT1, membrane fatty acid transporter (FAT/CD36), peroxisome proliferator activated receptor α (PPARα) and uncoupling protein 2 (UCP2) were analyzed. Results show that: (a) the hearts of SRD-fed rats display lipotoxicity suggesting impaired myocardial lipid utilization; (b) Compared with the SRD group, dietary chia normalizes blood pressure; reverses/improves heart lipotoxicity, glucose oxidation, the increased protein mass level of FAT/CD36, and the impaired insulin stimulated FAT/CD36 translocation to the plasma membrane. The enhanced M-CPT1 activity is markedly reduced without similar changes in protein mass. PPARα slightly decreases, while the UCP2 protein level remains unchanged in all groups. Normalization of dyslipidemia and IR by chia reduces plasma fatty acids (FAs) availability, suggesting that a different milieu prevents the robust translocation of FAT/CD36. This could reduce the influx of FAs, decreasing the elevated M-CPT1 activity and lipid storage and improving glucose oxidation in cardiac muscles of SRD-fed rats.

## 1. Introduction

Metabolic syndrome (MS) is a complex metabolic disorder influenced by genetic and environmental factors [1]. In Western societies, the high increase of MS, including cardiovascular disease (CVD), seems to be due to changes in lifestyle (e.g., increased consumption of food high in refined sugar and decreased physical activities). CVD represents a major cause of premature death in Western countries [2]. Therefore, there is growing interest in identifying novel therapeutic approaches including a particular focus on nutrition and dietary interventions.

Cardiac energy metabolic shifts occur as a normal response to diverse physiological and dietary conditions and as a component of the pathophysiological processes that accompany heart disease. It is well established that insulin and fatty acids (FAs) are important modulators of cardiac substrate utilization [3]. In the heart, there is a fine-tuning of high rates of myocellular FAs uptake and of mitochondrial fatty acid oxidation. When the rate of FA delivery to the heart increases (e.g., diabetes, high fat feeding) it may cause a mismatch between FA uptake and oxidation leading to excessive intracellular storage of the bio-active lipid intermediates within the cardiomyocytes that could subsequently lead to cardiac dysfunction [4,5]. Accordingly, several studies have shown that lipids accumulate in the heart of diabetic animals [6,7]. Myocardial FA uptake is largely regulated by the membrane fatty acid transporter (FAT/CD36) [8]. Chiu *et al.* [9] showed that a myocardial lipid accretion due to an increase of fat uptake leads to myocyte apoptosis and cardiomyopathy. An enhanced long-chain acyl CoA (LC ACoA) uptake and channeling into triglycerides was observed in the heart of obese Zucker rats [10]. The accumulation of triglyceride is likely toxic to the myocardium and has been linked with insulin resistance (IR) and cardiac dysfunction [6,11]. Besides, it is generally acknowledged that dietary factors, among them FAs, up-regulated the transcription of genes encoding for proteins involved in cardiac FA transport and metabolism, most likely through the activation of peroxisome proliferator activated receptor α (PPARα) (e.g., expression of muscle-type carnitine palmitoyltransferase 1 (M-CPT1) [12,13].

On the other hand, high sucrose, high fructose and/or high fat diets have been used to induce metabolic and physiological alterations in rodents, mimicking several aspects of the MS in humans such as dyslipidemia, IR and adiposity [14]. Furthermore, we have previously demonstrated that the cardiac muscle of rats chronically fed a sucrose-rich diet (SRD) showed a significant increase of lipid storage accompanied by a significant reduction of basal and insulin stimulated glucose uptake and metabolism (isolated perfusion according to Langendorff’s recirculating mode), as well as in the activities of key enzymes involved in glucose metabolism [15,16].

Epidemiological data show that a high intake of *n*-3 polyunsaturated fatty acids (*n*-3 PUFAs) from fish is associated with a lower incidence of heart failure and cardio protective function [17]. Moreover, different epidemiological and clinical studies have suggested that a high concentration of dietary α-linolenic acid 18:3 *n*-3, (ALA) is associated with a decreased risk of CVD [18,19]. Recent studies carried out in rats by Folino *et al.* [20] showed that ALA protects against cardiac injury and remodeling induced by beta-adrenergic over stimulation, and that a protective role is played by β_2_ adrenergic receptors which mediate the activation of the Src kinase-phosphatidylinositol-3-kinase protective pathway. The seeds of *Salvia* *hispanica* L, commonly known as chia seeds, contain the richest botanical oil source of ALA and high amounts of fiber and minerals. Poudyal *et al.* [21,22] have recently shown that the administration of chia oil improved heart left ventricular dimensions, contractility, volume and stiffness as well as hypertension, glucose tolerance and insulin sensitivity in rats fed a high fat-high fructose diet. In this line, recent studies of our group have demonstrated that the administration of chia seeds as a dietary source of fat in rats fed an SRD reversed dyslipidemia and IR, improved adipose tissue dysfunction and glucose and lipid metabolism in the skeletal muscle [23,24,25]. However, the effect of chia seeds on myocardial substrate utilization has been only partially investigated in this experimental model [21,22].

Thus, the aims of the present study were the following: (i) to further explore the mechanisms underlying the impaired lipid metabolism in the heart muscle of dyslipidemic insulin-resistant rats fed an SRD; (ii) to investigate if chia seeds as a dietary intervention could improve or even revert cardiac lipotoxicity. To achieve these goals: (a) we analyzed the protein mass levels of FAT/CD36 both at basal conditions and under insulin stimulation and the mitochondrial oxidation of LC ACoA by the activity and protein mass levels of the enzyme M-CPT1; (b) since the effect of FAs or FA derivatives in cardiac myocytes are considered to be PPARα mediated, we measured the protein mass level of this receptor; (c) we evaluated the protein mass levels of uncoupling protein 2 (UCP2), which plays a major role in the mitochondrial FAs flux. Additionally, the activities of the pyruvate dehydrogenase complex (PDHc) and lipid storage were assessed. The study was conducted in rats fed an SRD during 6 months, during which permanent dyslipidemia, IR, abnormal glucose homeostasis and visceral adiposity were present before the source of dietary fat, corn oil (CO), was replaced by an isocaloric amount of chia seeds for the last three months of the experimental period in half of the animals.

## 2. Materials and Methods

### 2.1. Animals

Male Wistar rats initially weighing 180–190 g purchased from the National Institute of Pharmacology (Buenos Aires, Argentina) were housed in an animal room under controlled temperature (22 ± 1 °C), humidity and airflow, with a fixed (12 h) light–dark cycle (lights on from 07:00 to 19:00 h) and with free access to water and food. Adequate measures were taken to minimize the pain or discomfort of the rats and we used the smallest number of animals as possible. The animal protocols were evaluated and approved by the Human and Animal Research Investigation Committee of the School of Biochemistry, University of Litoral, Argentina (FONCyT-PICT #945/2012).

### 2.2. Experimental Design

The rats (*n* = 60) were initially fed with a standard non-purified diet (Ralston Purina, St Louis, MO, USA). After one week of acclimation, the rats were randomly divided into two separate groups and were fed a semi-synthetic diet. The control group (*n* = 20) received a control diet (CD) containing corn starch (60% energy), protein (17% energy) and CO as sources of fat (23% energy) throughout the experimental period (6 months). The other group (*n* = 40) received the same semi-synthetic diet with the sucrose as the carbohydrate source (SRD). After 3 months, the animals in the SRD group were randomly divided into two subgroups. The rats in the first subgroup continued with the SRD diet for up to 6 months of feeding. The second subgroup received the Salba seed (chia) as the source of dietary fat (SRD + chia) for the next 3 months. The carbohydrates, proteins, fibers, vitamins and mineral contents in the chia seed of SRD + chia group were balanced with the CD and SRD groups, according to the amount of these nutrients present in the chia seeds. A detailed composition of each diet is described in Table 1. The fatty acid composition of each experimental diet is shown in Table 2. The preparation and handling of diets have been reported elsewhere [23,24]. All diets provided approximately 17 kJ/g of food.

**Table 1:** Composition of experimental diets **^1^**.

Diet Ingredients	Control Diet (CD)		Sucrose-Rich Diet (SRD)		SRD+chia Seed (SRD+chia)	
	% *w*/*w*	% Energy	% *w*/*w*	% Energy	% *w*/*w*	% Energy
Carbohydrates						
Corn starch	58.0	60.0	2.5	2.6	--	--
Sucrose	--	--	55.5	57.4	55.5	57.4
Chia seed **^2^**	--	--	--	--	2.5	2.6
Fat						
Corn oil	10.5	23.0	10.5	23.0	0.1	0.2
Chia seed	--	--	--	--	10.4	22.8
Protein						
Casein (vitamin free)	16.3	17.0	16.3	17.0	8.6	9.0
Chia seed	--	--	--	--	7.7	8.0

**^1^** The compositions of experimental diets are based on the AIN-93M diet. All diets contain by weight: salt mix 3.5% (AIN-93Mx); vitamin mix 1% (AIN-93Vx); choline chloride 0.2%; methionine 0.3%; fiber 10%–11%; **^2^** Chia seed (variety Salba; Salvia *hispánica* L.): 362 g/Kg diet. Chia composition (g/100 g chia seed): carbohydrate 37.45; insoluble fiber 81% of total of carbohydrates; fat 30.23; protein 21.19. Mineral composition (mg/100 g chia seed): Na 103.15; K 826.15; Ca 589.60; Fe 11.90; Mg 77.0; P 604.0; Zn 5.32; Cu 1.66; Mn 1.36.

**Table 2:** Total fatty acid composition of experimental diets (g/kg diet).

Fatty Acids ^1^	CD and SRD	SRD+chia Seed
16:0	10.92	6.96
18:0	2.73	2.42
18:1 *n*-9	33.71	7.39
18:2 *n*-6	54.10	19.85
18:3 *n*-3	0.80	67.26
20:1 *n*-9	0.47	0.36
Total saturated	13.65	9.38
Monounsaturated	34.18	7.75
Polyunsaturated		
*n*-6	54.10	19.85
*n*-3	0.80	67.26
*n*-6/*n*-3	67.62	0.295

**^1^** Other minor fatty acids have been excluded.

The body weight and energy intake of each animal were recorded twice per week throughout the experimental period in all groups and subgroups of rats. In a separate experiment, the individual caloric intake and weight gain of eight animals in each group and subgroup were assessed twice a week. At the end of the experimental period, food was removed at 07:00 h (end of the dark period) and unless otherwise indicated, experiments were performed under feed conditions.

### 2.3. Analytical Methods

Rats from the three dietary groups were anaesthetized with intraperitoneal sodium pentobarbital (60 mg/kg body weight). Blood samples were obtained from the jugular vein, collected in tubes containing sodium EDTA as anticoagulant and rapidly centrifuged. Plasma was either immediately assayed or stored at −20 °C. Plasma triglycerides, cholesterol, glucose and free fatty acids and immunoreactive insulin were determined as previously described [25]. Epididymal, retroperitoneal and omental adipose tissues were removed and weighed. The visceral adiposity index (%) was calculated as shown elsewhere [25]. The heart muscle was totally removed; then, it was weighed and the left ventricle was separated. The heart tissue was immediately frozen and stored at temperature of liquid N_2_. Heart weight was normalized relative to the tibia length at the time of removal. The homogenate of frozen muscle powder was used to determine triglyceride [26], LC ACoA [27] and diacylglycerol (DAG) content [28].

### 2.4. Determination of Blood Pressure

Blood pressure was measured in the three dietary groups in conscious animals maintained at 28 °C in a restraining tube, at the beginning, at 3 months and at the end of the experimental period using a CODA™ Monitor of tail-cuff non-invasive blood pressure system (Kent Scientific Corporation, Torrington, CT, USA). The values are reported as the average of 8 individual measurements.

### 2.5. Enzymatic Activity Assays of CPT and PDHc

Heart M-CPT1 enzyme activities were measured as reported by Ling *et al.* [29] with some modifications. Frozen tissue was homogenized in buffer (20 mM HEPES pH 7.4, 140 mM KCl, 10 mM EDTA and 5 mM MgCl_2_) and after centrifugation; the mitochondrial pellet was resuspended in a homogenization buffer. Protein concentration was measured by the Bradford assay (Bio-Rad reagent). To determine the total CPT activity, 100 µg of protein were added in a reaction buffer (20 mM HEPES pH 7.4, 220 mM sucrose, 40 mM KCl, 1 mM EGTA, 0.1 mM 5,5´-dithio-bis(2-nitrobenzoic acid) (DTNB) and 40 µM palmitoyl CoA). In addition, 1 mM of l-carnitine was added to start the reaction. The rate of appearance of CoASH-DTNB was monitored at 412 nm at 37 °C. CPT2 activity was measured under the same conditions in the presence of 10 µM of malonyl CoA (M-CPT1 inhibitor). The M-CPT1 activity was calculated as the difference between total CPT and CPT2 activities. The M-CPT1 activity was expressed as the amount of CoASH released per min per mg of protein. The extract and assay of the PDHc activity from the heart muscle were measured as previously described [15].

### 2.6. Determination of FAT/CD36 Protein Mass Level (Euglycemic-Hyperinsulinemic Clamp Studies)

The heart muscles of six rats from the CD, SRD and SRD+chia groups were rapidly removed (time 0 of clamp study) and stored at −80 °C for the determination of the FAT/CD36 protein levels. Immediately, in the other six rats from each dietary group, an infusion of highly purified porcine neutral insulin (Actrapid, Novo Nordisk,) was administered at 0.8 units/(kg × h) for 120 min. Glycemia was maintained at a euglycemic level by infusing glucose at a variable rate. The glucose infusion rate (GIR) during the second hour of the clamp study was taken as the net steady state of the whole body glucose. At the end of the clamp period, the heart muscle of each dietary group was rapidly removed for determination of the FAT/CD36 protein mass levels (for details see ref. [30]). Plasma membrane fractions from heart muscle were prepared according to the method of Rodnick *et al.* [31]. Briefly, the muscle was homogenized in ice-cold buffer (20 mM HEPES pH 7.4, 1 mM EDTA, 250 mM sucrose and 5 μL/mL protease inhibitor cocktail (Sigma, St. Louis, MO, USA)) and the homogenate was centrifuged at 3000× *g* at 4 °C. The supernatant was centrifuged at 184,000× *g* at 4 °C. The resulting pellet (membrane fraction) was resuspended in buffer and stored at −80 °C until the assay. Protein concentrations were quantified by the Bradford assay (Bio-Rad reagent). Proteins were separated by SDS-PAGE and transferred to PVDF membranes. The membranes were probed with a specific antibody (rabbit polyclonal anti-CD36 from Santa Cruz Biotechnology, Inc., Santa Cruz, CA, USA). The blot was incubated with horseradish peroxidase-linked secondary antibody (Santa Cruz Biotechnology) followed by chemiluminescence detection according to the manufacturer’s instruction (Pierce Biotechnology, Rockford, IL, USA). The protein levels were normalized to actin. The intensity of the bands was quantified by National Institutes of Health (NIH) imaging software (Bethesda, MD, USA). The relationship between the amount of the sample subjected to immunoblotting and the signal intensity observed was linear under the conditions described above.

### 2.7. Determination of M-CPT1 and PPARα Protein Mass Levels

Frozen heart powder was homogenized in a lysis buffer (10 mM Tris-HCl pH 7.4, 150 mM NaCl, 1 mM EGTA, 1 mM EDTA, 1% Triton X-100 and 5 μL/mL protease inhibitor cocktail (Sigma)) as described by Bogazzi *et al.* [32]. After 30 min incubation on ice, the lysates were centrifuged at 4 °C and supernatants were stored at −80 °C. The protein content was quantified by the Bradford assay. Total protein samples (100 μg/lane) were resolved on SDS-PAGE, transferred to PVDF membranes and probed with specific antibodies (rabbit polyclonal anti M-CPT1 or rabbit polyclonal anti-PPARα from Santa Cruz Biotechnology, Inc.). The blots were incubated with horseradish peroxidase-linked secondary antibody (Santa Cruz Biotechnology) followed by chemiluminescence detection as described above. The protein levels were normalized to actin.

### 2.8. Determination of UCP2 Protein Mass Level

The mitochondrial fraction from the heart muscle was carried out according to the method described by Pecqueur *et al.* [33]. Briefly, the tissue was homogenized at 4 °C in a TES buffer (10 mM Tris pH 7.5, 1 mM EDTA, 250 mM sucrose and 5 μL/mL protease inhibitor cocktail (Sigma)). After centrifugation, the mitochondrial pellet was resuspended in TES buffer and stored at −80 °C. The protein content was quantified by the Bradford assay. Protein samples (50 μg protein/lane) were resolved by SDS-PAGE and transferred to PVDF membranes. The membranes were probed with a specific antibody (goat polyclonal anti-UCP2 from Santa Cruz Biotechnology, Inc.). The blot was incubated with horseradish peroxidase-linked secondary antibody (Santa Cruz Biotechnology) followed by chemiluminescence detection as described above. The protein levels were normalized to actin.

### 2.9. Statistical Analysis

Sample sizes were calculated on the basis of measurements previously made with rats fed either a CD or SRD [16,25,30] considering an 80% power as described by Glantz [34]. Results were expressed as mean with their standard errors. The homogeneity of variances were tested using Barlett’s test. The statistical significance between groups was determined by one way analysis of variance (ANOVA) with one factor (diet) followed by the Newman Keul`s multiple comparison *post hoc* test [35]. Differences having *p* values lower than 0.05 were considered to be statistically significant. Statistical analyses were performed using GraphPad Prism version 5.00 for Windows (San Diego, CA, USA). All reported *p* values are 2-sided.

## 3. Results

### 3.1. Body Weight, Energy Intake and Visceral Adiposity Index

Body weight and energy intake were carefully monitored in all groups of rats through the experimental period. As previously shown [25], a significant increase in body weight and energy intake occurred in rats fed an SRD from month 3 to 6 compared to those fed a CD (Table 3). A similar energy intake was recorded in both SRD and SRD+chia groups during the last three months of the experimental period while body weight at month 6 was slightly lower without statistical differences in the latter group. As in our previous report [24], SRD-fed rats showed a significant increase of the visceral adiposity index compared to the CD group. The chia seed enriched diet significantly reduced the aforementioned index, which reached values similar to those of the CD group.

**Table 3:** Body weight, energy intake and adiposity index of rats fed a control diet (CD), sucrose-rich diet (SRD) or SRD + chia seed (SRD + chia) **^1^**.

Diet	Body Weight (g)		Energy Intake (kJ/Day)	Diet	Body Weight (g)	Energy Intake (kJ/Day)	Visceral Adiposity Index (%)
	Initial	3 Months	Initial to 3 Months		6 Months	3 to 6 Months	6 Months
CD (8)	184.3 ± 2.6	414.5 ± 5.5	294.5 ± 12.5	CD (8)	476.3 ± 7.6 ^b^	292.0 ± 7.2 ^b^	4.1 ± 0.3 ^b^
SRD (16)	186.0 ± 1.6	428.0 ± 6.0	292.0 ± 7.2	SRD (8)	545.0 ± 10.0 ^a^	374.0 ± 9.5 ^a^	6.2 ± 0.4 ^a^
				SRD+chia (8)	524.0 ± 7.3 ^a^	356.5 ± 13.0 ^a^	4.5 ± 0.2 ^b^

**^1^** Values are expressed as mean ± SEM, () number of rats. Values in a column that do not share the same superscript letter (a, b) are significantly different *p* < 0.05 when one variable at a time was compared by the Newman Keul`s test.

### 3.2. Total and Relative Heart Weight and Systolic Blood Pressure

Total heart weights recorded at the end of experimental period in each dietary group showed a significant increase in SRD-fed rats compared with the CD-fed rats. SRD + chia did not modify heart weight. Conversely, relative heart weights as g/100 g body weight or by the length of the tibia (mg/mm) were similar in all dietary groups (Table 4). SRD feeding increased systolic blood pressure throughout the six-month feeding protocol with values at three and six months significantly higher compared to those observed in the CD-fed group. Chia seeds in the SRD group normalized systolic blood pressure after three months of treatment reaching values similar to those of the CD group (Table 4).

**Table 4:** Total and relative heart weight at the end of the experimental period and systolic blood pressure of rats fed a control diet (CD), sucrose-rich diet (SRD) or SRD + chia seed (SRD + chia) **^1^**.

	CD	SRD	SRD+chia
Heart tissue			
Total wet weight, g	1.24 ± 0.01 ^b^ (8)	1.31 ± 0.03 ^a^ (8)	1.28 ± 0.02 ^a^ (8)
g wet weight/100 g body weight	0.260 ± 0.003 (8)	0.250 ± 0.004 (8)	0.250 ± 0.005 (8)
mg wet weight/mm tibia length	27.2 ± 1.0 (8)	30.1 ± 1.3 (8)	28.3 ± 1.0 (8)
Systolic blood pressure, mmHg			
Initial	115.0 ± 4.6 (8)	116.0 ± 2.4 (16)	
3 months	123.3 ± 1.0 ^b^ (8)	130.9 ± 1.2 ^a^ (16)	
6 months	120.2 ± 4.8 ^b^ (8)	138.4 ± 1.6 ^a^ (8)	118.8 ± 1.6 ^b^ (8)

**^1^** Values are expressed as mean ± SEM, () number of rats. Values in a line that do not share the same superscript letter (a, b) are significantly different *p* < 0.05 when one variable at a time was compared by the Newman Keul`s test.

### 3.3. Plasma Metabolites, Insulin Levels and GIR

**Figure 1:**
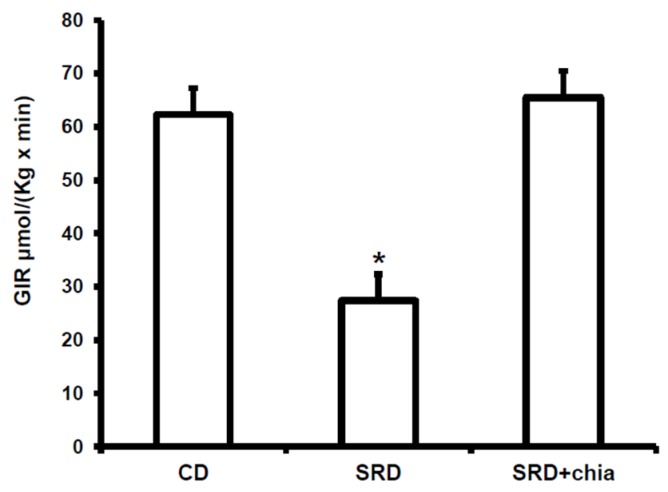
GIR during euglycemic-hyperinsulinemic clamp in rats fed a control diet (CD), sucrose-rich diet (SRD) or SRD + chia seed (SRD + chia).

Confirming previous studies [25], the changes observed after three or six months of SRD on plasma triglyceride, free fatty acids and glucose concentration were similar and both significantly different from those of the CD groups to which the data from SRD + chia were similar (Table 5). No statistical differences in plasma insulin levels were observed at the end of the experimental period between the three dietary groups. Furthermore, the significant decrease of the GIR recorded in the SRD-fed group returned to values similar to those obtained in the CD-fed rats in the SRD + chia group (Figure 1).

**Table 5:** Plasma metabolites of rats fed a control diet (CD), sucrose-rich diet (SRD) or SRD + chia seed (SRD+chia) **^1^**.

Diet	Time on Diet (Months)	Triglyceride (mM)	Free Fatty Acids (μM)	Cholesterol (mM)	Glucose (mM)	Insulin (μU/mL)
CD	3	0.69 ± 0.04 ^b^	300.1 ± 16.0 ^b^	1.85 ± 0.10 ^b^	6.5 ± 0.2 ^b^	64.1 ± 3.2
SRD	3	1.98 ± 0.08 ^a^	716.0 ± 8.1 ^a^	3.21 ± 0.14 ^a^	7.9 ± 0.1 ^a^	60.1 ± 4.2
CD	6	0.72 ± 0.03 ^b^	335.0 ± 13.0 ^b^	1.92 ± 0.11 ^b^	6.6 ± 0.1 ^b^	62.0 ± 2.9
SRD	6	2.06 ± 0.17 ^a^	760.4 ± 16.3 ^a^	3.60 ± 0.04 ^a^	8.3 ± 0.1 ^a^	65.0 ± 3.2
SRD+chia	3 to 6	0.72 ± 0.05 ^b^	363.0 ± 35.4 ^b^	1.75 ± 0.21 ^b^	6.9 ± 0.1 ^b^	67.4 ± 6.5

**^1^** Values are expressed as mean ± SEM, *n* = 6. Values in a column that do not share the same superscript letter (a, b) are significantly different *p* < 0.05 when one variable at a time was compared by the Newman Keul`s test.

Values are mean, with their standard errors depicted by vertical bars (six animals per group).* *p* < 0.05 SRD *vs.* CD and SRD + chia.

### 3.4. Heart Muscle Metabolites Concentrations and PDHc Activity

Table 6 depicts significant increases of lipid storage (triglycerides, LC ACoA and DAG) levels within the cardiac muscle of SRD-fed rats. The present data show that in the SRD + chia seed group, neither parameter differed from those of the CD group at the end of the experimental period. Moreover, the administration of chia seed (SRD+chia) was able to revert the reduced active form of PDHc (PDHa) observed in the SRD-fed group reaching values similar to those of the control group (CD). The total PDHc activity expressed per gram of wet tissue did not differ between the groups (data not shown).

**Table 6:** Intramyocardial lipid accumulation and PDHc activity of rats fed a control diet (CD), sucrose-rich diet (SRD) or SRD + chia seed (SRD + chia) at the end of the experimental period **^1^**.

	CD	SRD	SRD+chia
Triglyceride (μmol/g wet tissue)	3.60 ± 0.22 ^b^	6.03 ± 0.34 ^a^	4.44 ± 0.60 ^b^
LC ACoA (nmol/g wet tissue)	31.4 ± 5.5 ^b^	68.0 ± 4.0 ^a^	45.8 ± 5.5 ^b^
DAG (nmol/g wet tissue)	250.8 ± 19.5 ^b^	355.3 ± 15.4 ^a^	276.6 ± 24.0 ^b^
PDHa (% of total PDHc)	59.7 ± 5.8 ^a^	26.6 ± 4.6 ^b^	45.1 ± 4.9 ^a^

**^1^** Values are expressed as mean ± SEM, *n* = 6. Values in a line that do not share the same superscript letter (a, b) are significantly different *p* < 0.05 when one variable at a time was compared by the Newman Keul`s test.

### 3.5. FAT/CD36 Protein Mass Level in Heart Muscle at the Beginning and at the End of the Euglycemic-Hyperinsulinemic Clamp Studies

**Figure 2:**
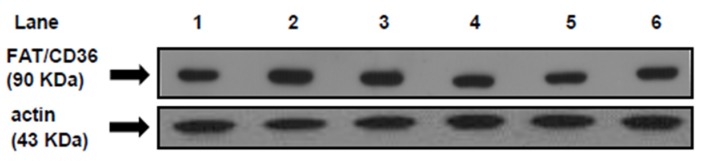

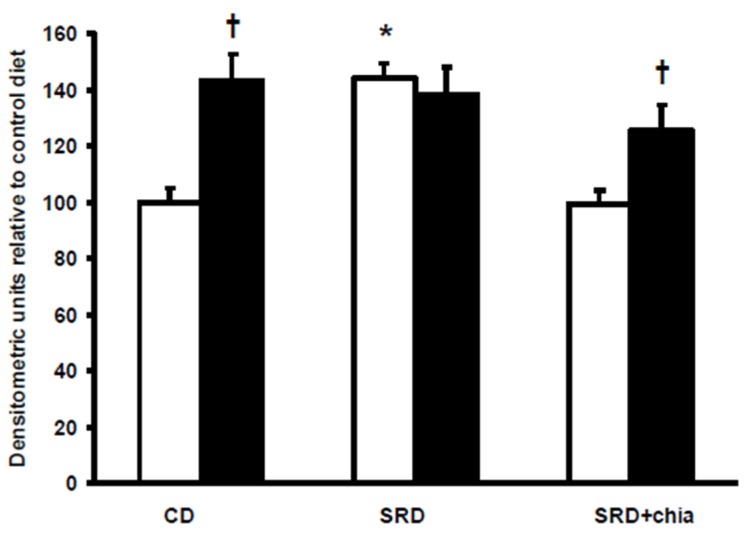
Heart protein mass levels of FAT/CD36 at the beginning (0 min) and under the insulin stimulation at the end (120 min) of the clamp studies in rats fed a control diet (CD), a sucrose-rich diet (SRD) or SRD + chia seed (SRD + chia). Top panel: a representative immunoblot of heart FAT/CD36 of rats fed CD, SRD and SRD + chia. Molecular marker is shown on the right. Lane 1, CD 0 min; lane 2, CD 120 min; lane 3, SRD 0 min; lane 4, SRD 120 min; lane 5, SRD + chia 0 min; lane 6, SRD + chia 120 min. Bottom panel: densitometric immunoblot analysis of heart FAT/CD36 protein mass level of rats fed CD, SRD or SRD + chia at the beginning (0 min □) and at the end (120 min ■) of clamp studies.

Figure 2 shows the protein mass level of FAT/CD36 at the beginning (0 min) and at the end of the clamp. The immunoblottting of the heart muscle revealed a single 90 KDa band consistent with FAT/CD36. Each gel contained an equal number of samples from rats fed a CD, SRD and SRD + chia at 0 min and 120 min of euglycemic hyperinsulinemic clamp (Figure 2, top panel). After the densitometry of immunoblots, FAT/CD36 at the beginning of the clamp was normalized to 100%, and both the SRD and SRD + chia groups at the beginning as well as the three dietary groups at the end of the study were expressed relative to this. At the beginning of the clamp, the qualitative and quantitative analysis of the Western blot showed a significant increase (*p* < 0.05) in the relative abundance of FAT/CD36 in the SRD-fed rats compared to the CD and SRD+chia groups. The latter reached values similar to those of the CD group. At the end of the clamp study, insulin stimulated the translocation of FAT/CD36 to the sarcolemma in the CD-fed rats that showed a significant (*p* < 0.05) increase of their protein mass level, while in the heart of SRD-fed rats, insulin did not further recruit FAT/CD36 to the sarcolemma under the same experimental conditions. Moreover, changes induced by the SRD were reverted by the chia seed enriched diet (Figure 2 bottom panel).

Values are mean, with their standard errors depicted by vertical bars (six animals per group) and expressed as percentage relative to the control diet at 0 min of the clamp. * *p* < 0.05 SRD at 0 min *vs*. CD and SRD+chia at 0 min of the clamp. † *p* < 0.05 CD and SRD+chia at 120 min *vs*. CD and SRD+chia at 0 min of the clamp.

### 3.6. M-CPT1 Activity and Protein Mass Level

**Figure 3:**
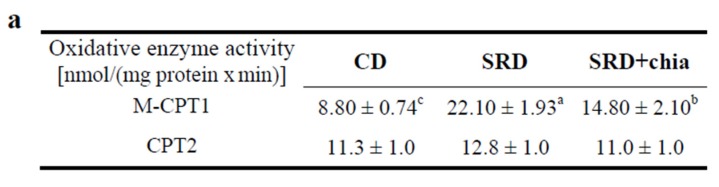

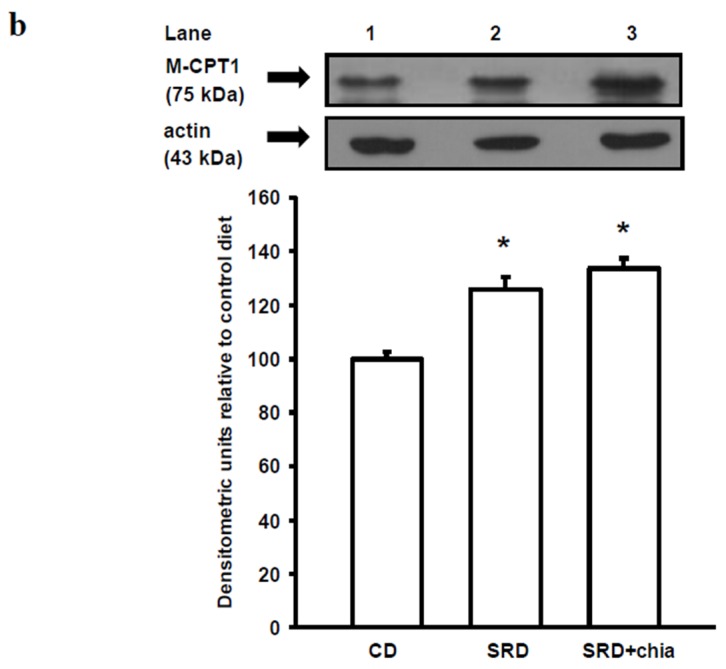
(**a**) Heart enzyme activities of M-CPT1 and CPT2 in rats fed a control diet (CD), sucrose-rich diet (SRD) or SRD+chia seed (SRD+chia). Values are expressed as mean ± SEM (six animals per group). Values in a line that do not share the same superscript letter (**a**, **b**, **c**) are significantly different (*p* < 0.05) when one variable at time was compared by the Newman Keul`s test; (**b**) Top panel: a representative immunoblot of heart M-CPT1 protein mass level of rats fed CD, SRD and SRD + chia. Molecular marker is shown on the right. Lane 1, CD; lane 2, SRD; lane 3, SRD + chia. Bottom panel: densitometric immunoblot analysis of heart M-CPT1 protein mass level of rats fed CD, SRD or SRD + chia.

Figure 3a shows the cardiac muscle activity of the M-CPT1 in the three dietary groups. Compared with the CD-fed group, a three-fold increase of the mitochondrial M-CPT1 activity was observed in the heart of rats fed an SRD. The M-CPT1 activity was significantly reduced under the administration of chia seed. However, values were still higher than those recorded in the CD-fed rats. The CPT2 activity remained similar in the three dietary groups. Immunoblotting of cardiac muscle M-CPT1 revealed a single 75 KDa band consistent with M-CPT1. Each gel contained an equal number of samples from rats fed a CD, SRD and SRD+chia (Figure 3b top panel). After densitometry of immunoblots the M-CPT1 of the CD group was normalized to 100%, and both the SRD and SRD+chia groups were expressed relative to this. The qualitative and quantitative abundance of the Western blot showed that M-CPT1 protein mass level significantly increased (*p* < 0.05) in the heart muscle of the SRD group when compared with rats fed a CD (Figure 3b bottom panel). Interestingly, although the enzymatic activity of M-CPT1 decreased when chia seed replaced CO as a source of fat in the SRD, the relative abundance of M-CPT1 was still significantly higher.

Values are mean, with their standard errors depicted by vertical bars (six animals per group) and expressed as percentage relative to the control diet. * *p* < 0.05 SRD and SRD + chia *vs*. CD.

### 3.7. PPARα Protein Mass Level

**Figure 4:**
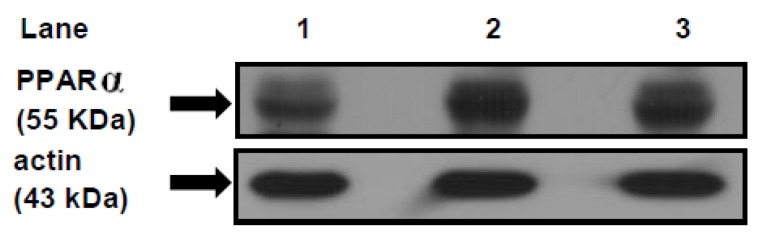

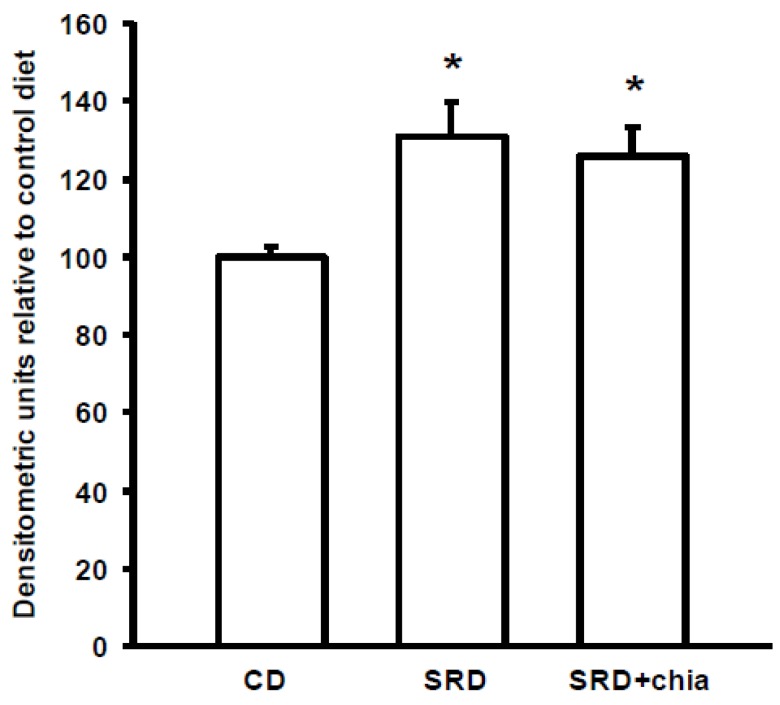
Heart protein mass level of PPARα in rats fed a control diet (CD), sucrose-rich diet (SRD) or SRD + chia seed (SRD + chia). Top panel: a representative immunoblot of heart PPARα of rats fed a CD, SRD and SRD + chia. Molecular marker is shown on the right. Lane 1 CD; lane 2 SRD; lane 3 SRD + chia. Bottom panel: densitometric immunoblot analysis of heart PPARα protein mass level of rats fed CD, SRD or SRD + chia.. Values are mean, with their standard errors depicted by vertical bars (6 animals per group) and expressed as percentage relative to the control diet. * *p* < 0.05 SRD and SRD + chia *vs.* CD.

We examined the protein mass level of PPARα, since this receptor is considered a master regulator of FAs metabolism in several organs including the heart. The immunoblotting of the cardiac muscle revealed a single 55KDa band consistent with PPARα. Each gel contained an equal number of samples from the CD, SRD and SRD + chia groups (Figure 4 top panel). After the densitometry of immunoblots, the PPARα of the CD group was normalized to 100%, and both the SRD and SRD + chia groups were expressed relative to this. The qualitative and quantitative abundance of the Western blot showed that the relative abundance of the PPARα protein mass level significantly increased (*p* < 0.05) in the hearts of both the SRD and SRD + chia groups, although the protein mass level was slightly lower without statistical differences in the latter group (Figure 4 bottom panel). On the other hand, we measured the protein mass levels of UCP2 since this uncoupling protein could be regulated by increased FA concentration through PPARα activation. The qualitative and quantitative analysis of the Western blots showed that the relative abundance of mitochondrial UCP2 protein mass level was similar in the hearts of the three dietary groups. Values were as follows: (mean ± SEM, *n* = 6); CD 100 ± 3.2; SRD 103.8 ± 2.5 and SRD + chia 95.7 ± 5.3, p NS.

## 4. Discussion

The present study provides new information on the mechanisms involved in heart muscle lipotoxicity in dyslipemic insulin resistant rats fed an SRD and explores the possible beneficial effects of dietary chia supplementation on reversed or improved pre-exiting impaired cardiac lipid metabolism. Disruption of the sensitive balance between FAs and glucose in the heart and increased intramyocellular fat contents and fatty acid metabolites are likely to play a pivotal role in the development of insulin resistance, cardiac lipotoxicity and heart dysfunction [36]. FAT/CD36 plays a pivotal role in governing myocardial FAs uptake [8]. In the present work, the increased intracellular LC ACoA in the heart of SRD-fed rats is accompanied by a significant increase of protein mass level of FAT/CD36 in the plasma membrane, suggesting that the enhanced amount of FAT/CD36 on sarcolemma elicits an increased rate of FAs uptake. In this regard, an increased availability of plasma free fatty acids and triglyceride levels is recorded in the SRD-fed group. Moreover, despite a significant increase of both the M-CPT1 activity and its protein mass level, triglyceride accumulates in the heart of this dietary group. It is possible that the increased flux of FAs to the heart exceeds the mitochondrial oxidative capacity leading to an increase of FAs storage into the triglyceride pool. The dynamic equilibrium between triglyceride stores and their metabolites cause accumulation of DAG and ceramide during prolonged long-chain fatty acid (LCFA) influx. Although in the present study we did not measure the level of ceramide, our previous results [16] and the present data show an increase of DAG concentration in the heart of rats fed an SRD. Both metabolites are implicated in counteracting insulin signaling, reducing insulin responsiveness and altering its ability to regulate substrate handling [4]. In this regard, the present data show that insulin stimulated the cell surface recruitment of FAT/CD36 in the heart of CD-fed rats. However, insulin was unable to further recruit FAT/CD36 to the sarcolemma in the heart of SRD-fed rats that was completely insensitive to the stimulus of the hormone. Similarly, in cardiac myocytes from obese Zucker rats, Coort *et al.* [10] reported that insulin failed to alter the sub cellular localization of FAT/CD36 and the rate of LCFA uptake and triglyceride esterification. Besides, in cardiamyocytes of Wistar rats in which a high-fat diet induced cardiac contractile dysfunction, Ouwens *et al.* [5] demonstrated that a permanent presence of FAT/CD36 in the sarcolemma membrane resulted in the enhancement of LCFA uptake and myocardial triglyceride accumulation.

PPARα and its co-activator PPARγ co-activator 1 alpha (PGC-1α) play an important role in the transcriptional regulation of cardiac energy metabolism, and the effect of FAs in cardiac myocytes is considered to be PPARα mediated [37]. Several lines of evidence suggested that LCFAs that induce the gene expression of M-CPT1 and other enzymes in the cellular fatty acid utilization pathway are namely mediated by PPARα transcriptional control [13]. LCFAs: linoleic acid 18:2 *n*-6, ALA and docosahexaenoic acid 22:6, *n*-3 (DHA) among them, and a variety of related compounds serve as PPARα ligands [38]. In this context, the present study shows a significant increase of the relative abundance of the protein mass level of PPARα and the mitochondrial M-CPT1 activity in the heart of SRD-fed rats. These results suggest that a chronic high exposure to Fas, which enhances their uptake, induces the activation of PPARα protein expression that, in turn, encodes the proteins responsible for FAs oxidation, M-CPT1 among others. Since an increase of intramyocardial lipids is observed in the SRD heart, it is possible that a disruption of the balance between lipid oxidation-storage occurs in the heart muscle of this dyslipemic insulin-resistant model. In this vein, Buchanam *et al.* [39] have documented an increased PPARα and PGC-1α expression in murine insulin-resistant hearts. A high fat diet also activates PPARα in the heart and stimulates expression of key proteins involved in fatty acid oxidation [40].

It is well known that dietary *n*-3 PUFAs, mainly eicosapentaenoic acid 20:5, *n*-3 (EPA) and DHA, improve cardiac function [17]. ALA could be a valuable source of *n*-3 long-chain FAs via elongase/desaturase activities. Dietary ALA exerts a protective effect on the CVD [18]. In this regard, and confirming previous results, the present work demonstrates a reversion of dyslipidemia, abnormal glucose homeostasis and whole body peripheral insulin insensitivity when dietary chia seed replaced CO in the SRD-fed rats. The reverse of dyslipidemia by chia seed led to a significant reduction of lipid storage in the heart of SRD-fed rats, reaching values similar to those observed in the heart of CD-fed rats. Moreover, at basal conditions (beginning of the clamp study) a decrease of FAT/CD36 protein mass level suggests that a different milieu (decreased plasma lipids levels) prevented the robust relocations of fatty acid translocase to the sarcolemma, which was otherwise seen in the cardiac muscle of SRD-fed rats and, therefore, reduced the influx of FAs. Furthermore, the heart of dietary chia-fed rats was sensitive to the stimulus of insulin. As in the CD-fed group, the hormone significantly induced the translocation of FAT/CD36 to the plasma membrane. Recently, in isolated rat cardiomyocytes incubated under insulin resistance evoking conditions, Franekova *et al.* [41] demonstrated that the inclusion of EPA and DHA to the medium prevented the persistent translocation of CD36 to the sarcolemma and protected the metabolic and functional properties of the cardiomyocytes. In this regard, we previously demonstrated that the administration of dietary fish oil to SRD-fed rats was able to reverse heart muscle lipotoxicity and benefit key enzyme activities involved in the glucose metabolism [16]. At present, we are unaware of other studies concerning the effect of the long-term consumption of dietary chia and/or ALA on lipid metabolism in the cardiac muscle of SRD-fed rats. However, the reversion of the impaired glucose oxidation, as well as the accretion of triglyceride and fatty acid derivatives, the normalization of the enhanced sarcolemmal FAT/CD36 and the significantly reduced mitochondrial oxidative flux suggests that dietary chia seeds could improve the altered balance of heart fuel utilization. Interestingly, compared with CD-fed rats the protein mass level of the nuclear receptor PPARα and the activity of its target enzyme M-CPT1 were still higher in the heart of the SRD + chia group. Our results do not provide data on the mechanisms underlying the effect of chia seed on this nuclear receptor but it was shown that ALA and DHA, among others, are natural occurring ligands of PPARα in the heart [38]. In this regard, it has been demonstrated by our group [23] and others [21,42] that chia seeds change the plasma fatty acid profile increasing ALA, EPA, docosapentaenoic acid 22:5, *n*-3 (DPA) and DHA levels as well as the *n*-3/*n*-6 FAs ratio in rats fed an SRD, a control diet, or a high fat, high fructose diet. Thus, we do not discard the possibility that the different plasma FA profiles to which the heart was exposed could contribute to this finding.

On the other hand, an increase of FAs could induce UCP2 expression through PPARα activation in adult rat cardiomyocytes [43]. Besides, an increase of UCP2 and PPARα was recorded in the heart of ob/ob mice [39]. However, under the present experimental protocol, our results showed that the mitochondrial protein mass levels of UCP2 were similar in the three experimental groups. Besides, we are unaware of any other studies that evaluated the potential role of UCP2 in altering myocardial substrate in the heart muscle of SRD rats, and the effect of either chia seeds or oil upon UCP2. Further studies will be needed to evaluate this matter.

Chia seeds were able to decrease the systolic blood pressure that developed in the SRD-fed rats. In this regard, Poudyal *et al.* [21], in rats fed a high fructose-high fat diet, recorded that chia seed normalized systolic blood pressure increasing DPA and DHA contents in FAs of the heart, and Rousseau *et al.* [44] showed a decrease in blood pressure and increased DHA and EPA in cardiac phospholipids in rats fed a high fructose diet supplemented with either DHA or EPA. Interestingly, we observed an increase of DHA and ALA in the FA phospholipids of cardiac membrane in the SRD + chia group (data not shown). Moreover, Vuksan *et al.* [45] showed that a long-term supplementation with Salba (*S.* *hispanica* L) attenuates systolic blood pressure and emerging cardiovascular risk factors, safely beyond conventional therapy, while maintaining good glycemic and lipid control in well-controlled type-2 diabetic patients.

## 5. Conclusions

In brief, this study demonstrated that the lipotoxicity present in the heart of SRD-fed rats, an experimental model of dyslipidemia and insulin resistance, is accompanied by changes involving lipid metabolism suggesting an impaired myocardial lipid utilization. In this scenario, and for the first time, this work also provides new information concerning the possible mechanisms underlying the beneficial effects of dietary chia seeds on lipid cardiac metabolism and glucose oxidation. Although caution is warranted before extrapolating results from rodents to humans, chia seeds may serve as an alternative dietary strategy in the management of these metabolic alterations susceptible to dietary manipulation.

## References

[1] Pollex R.L., Hegele R.A. (2006). Genetic determinants of the metabolic syndrome. Nat. Clin. Pract. Cardiovasc. Med..

[2] Gaziano T.A., Bitton A., Anand S., Abrahams-Gessel S., Murphy A. (2010). Growing epidemic of coronary heart disease in low- and middle- income countries. Curr. Probl. Cardiol..

[3] Mazumder P.K., O’Neill B.T., Roberts M.W., Buchanan J., Yun U.J., Cooksey R.C., Boudina S., Abel E.D. (2004). Impaired cardiac efficiency and increased fatty acid oxidation in insulin-resistant Ob/Ob mouse hearts. Diabetes.

[4] Glatz J.F., Angin Y., Steinbusch L.K., Schwenk R.W., Luiken J.J. (2013). CD36 as a target to prevent cardiac lipotoxicity and insulin resistance. Prostaglandins Leukot. Essent. Fatty Acids.

[5] Ouwens D.M., Diamant M., Fodor M., Habets D.D., Pelsers M.M., El H.M., Dang Z.C., van den Brom C.E., Vlasblom R., Rietdijk A. (2007). Cardiac contractile dysfunction in insulin-resistant rats fed a high-fat diet is associated with elevated CD36-mediated fatty acid uptake and esterification. Diabetologia.

[6] Zhou Y.T., Grayburn P., Karim A., Shimabukuro M., Higa M., Baetens D., Orci L., Unger R.H. (2000). Lipotoxic heart disease in obese rats: Implications for human obesity. Proc. Natl. Acad. Sci. USA.

[7] Paulson D.J., Crass M.F. (1982). Endogenous triacylglycerol metabolism in diabetic heart. Am. J. Physiol..

[8] Van Oort M.M., van Doorn J.M., Bonen A., Glatz J.F., van der Horst D.J., Rodenburg K.W., Luiken J.J. (2008). Insulin-induced translocation of CD36 to the plasma membrane is reversible and shows similarity to that of GLUT4. Biochim. Biophys. Acta.

[9] Chiu H.C., Kovacs A., Ford D.A., Hsu F.F., Garcia R., Herrero P., Saffitz J.E., Schaffer J.E. (2001). A novel mouse model of lipotoxic cardiomyopathy. J. Clin. Investig..

[10] Coort S.L., Hasselbaink D.M., Koonen D.P., Willems J., Coumans W.A., Chabowski A., van der Vusse G.J., Bonen A., Glatz J.F., Luiken J.J. (2004). Enhanced sarcolemmal FAT/CD36 content and triacylglycerol storage in cardiac myocytes from obese Zucker rats. Diabetes.

[11] Finck B.N., Han X., Courtois M., Aimond F., Nerbonne J.M., Kovacs A., Gross R.W., Kelly D.P. (2003). A critical role for PPARalpha-mediated lipotoxicity in the pathogenesis of diabetic cardiomyopathy: Modulation by dietary fat content. Proc. Natl. Acad. Sci. USA.

[12] Barger P.M., Kelly D.P. (2000). PPAR signaling in the control of cardiac energy metabolism. Trends Cardiovasc. Med..

[13] Brandt J.M., Djouadi F., Kelly D.P. (1998). Fatty acids activate transcription of the muscle carnitine palmitoyltransferase I gene in cardiac myocytes via the peroxisome proliferator-activated receptor alpha. J. Biol. Chem..

[14] Lombardo Y.B., Chicco A.G. (2006). Effects of dietary polyunsaturated *n*-3 fatty acids on dyslipidemia and insulin resistance in rodents and humans. A review. J. Nutr. Biochem..

[15] Montes M., Chicco A., Lombardo Y.B. (2000). The effect of insulin on the uptake and metabolic fate of glucose in isolated perfused hearts of dyslipemic rats. J. Nutr. Biochem..

[16] D’Alessandro M.E., Chicco A., Lombardo Y.B. (2008). Dietary fish oil reverses lipotoxicity, altered glucose metabolism, and nPKCepsilon translocation in the heart of dyslipemic insulin-resistant rats. Metabolism.

[17] Jump D.B., Depner C.M., Tripathy S. (2012). Omega-3 fatty acid supplementation and cardiovascular disease. J. Lipid Res..

[18] Djousse L., Arnett D.K., Carr J.J., Eckfeldt J.H., Hopkins P.N., Province M.A., Ellison R.C. (2005). Dietary linolenic acid is inversely associated with calcified atherosclerotic plaque in the coronary arteries: The National Heart, Lung, and Blood Institute Family Heart Study. Circulation.

[19] Mozaffarian D., Ascherio A., Hu F.B., Stampfer M.J., Willett W.C., Siscovick D.S., Rimm E.B. (2005). Interplay between different polyunsaturated fatty acids and risk of coronary heart disease in men. Circulation.

[20] Folino A., Sprio A.E., di Scipio F., Berta G.N., Rastaldo R. (2015). Alpha-linolenic acid protects against cardiac injury and remodeling induced by beta-adrenergic overstimulation. Food Funct..

[21] Poudyal H., Panchal S.K., Ward L.C., Waanders J., Brown L. (2012). Chronic high-carbohydrate, high-fat feeding in rats induces reversible metabolic, cardiovascular, and liver changes. Am. J. Physiol. Endocrinol. Metab..

[22] Poudyal H., Panchal S.K., Ward L.C., Brown L. (2013). Effects of ALA, EPA and DHA in high-carbohydrate, high-fat diet-induced metabolic syndrome in rats. J. Nutr. Biochem..

[23] Chicco A.G., D’Alessandro M.E., Hein G.J., Oliva M.E., Lombardo Y.B. (2009). Dietary chia seed (Salvia *hispanica* L.) rich in alpha-linolenic acid improves adiposity and normalises hypertriacylglycerolaemia and insulin resistance in dyslipaemic rats. Br. J. Nutr..

[24] Rossi A.S., Oliva M.E., Ferreira M.R., Chicco A., Lombardo Y.B. (2013). Dietary chia seed induced changes in hepatic transcription factors and their target lipogenic and oxidative enzyme activities in dyslipidaemic insulin-resistant rats. Br. J. Nutr..

[25] Oliva M.E., Ferreira M.R., Chicco A., Lombardo Y.B. (2013). Dietary salba (Salvia *hispanica* L) seed rich in alpha-linolenic acid improves adipose tissue dysfunction and the altered skeletal muscle glucose and lipid metabolism in dyslipidemic insulin-resistant rats. Prostaglandins Leukot. Essent. Fatty Acids.

[26] Laurell S. (1966). A method for routine determination of plasma triglycerides. Scan. J. Clin. Lab. Investig..

[27] Lowenstein J.M. (1969). Citric Acid cycle. Methods in Enzymology.

[28] Schmitz-Peiffer C., Browne C.L., Oakes N.D., Watkinson A., Chisholm D.J., Kraegen E.W., Biden T.J. (1997). Alterations in the expression and cellular localization of protein kinase C isozymes ε and θ are associated with insulin resistance in skeletal muscle of the high-fat-fed rat. Diabetes.

[29] Ling B., Aziz C., Alcorn J. (2012). Systematic evaluation of key l-carnitine homeostasis mechanisms during postnatal development in rat. Nutr. Metab (Lond.).

[30] Chicco A., D’Alessandro M.E., Karabatas L., Pastorale C., Basabe J.C., Lombardo Y.B. (2003). Muscle lipid metabolism and insulin secretion are altered in insulin-resistant rats fed a high sucrose diet. J. Nutr..

[31] Rodnick K.J., Slot J.W., Studelska D.R., Hanpeter D.E., Robinson L.J., Geuze H.J., James D.E. (1992). Immunocytochemical and biochemical studies of GLUT4 in rat skeletal muscle. J. Biol. Chem..

[32] Bogazzi F., Raggi F., Ultimieri F., Russo D., D’Alessio A., Manariti A., Brogioni S., Manetti L., Martino E. (2009). Regulation of cardiac fatty acids metabolism in transgenic mice overexpressing bovine GH. J. Endocrinol..

[33] Pecqueur C., Alves-Guerra M.C., Gelly C., Levi-Meyrueis C., Couplan E., Collins S., Ricquier D., Bouillaud F., Miroux B. (2001). Uncoupling protein 2, *in vivo* distribution, induction upon oxidative stress, and evidence for translational regulation. J. Biol. Chem..

[34] Glantz S.A. (2005). Primer of Biostatistics.

[35] Snedecor G.W., Cochran W.G. (1967). Factorial Experiments, in Statistical Methods Applied to Experimental in Agriculture and Biology.

[36] Chess D.J., Stanley W.C. (2008). Role of diet and fuel overabundance in the development and progression of heart failure. Cardiovasc. Res..

[37] Duncan J.G. (2011). Peroxisome proliferator activated receptor-alpha (PPARalpha) and PPAR gamma coactivator-1alpha (PGC-1alpha) regulation of cardiac metabolism in diabetes. Pediatr. Cardiol..

[38] Georgiadi A., Boekschoten M.V., Muller M., Kersten S. (2012). Detailed transcriptomics analysis of the effect of dietary fatty acids on gene expression in the heart. Physiol. Genom..

[39] Buchanan J., Mazumder P.K., Hu P., Chakrabarti G., Roberts M.W., Yun U.J., Cooksey R.C., Litwin S.E., Abel E.D. (2005). Reduced cardiac efficiency and altered substrate metabolism precedes the onset of hyperglycemia and contractile dysfunction in two mouse models of insulin resistance and obesity. Endocrinology.

[40] Stanley W.C., Dabkowski E.R., Ribeiro R.F., O’Connell K.A. (2012). Dietary fat and heart failure: Moving from lipotoxicity to lipoprotection. Circ. Res..

[41] Franekova V., Angin Y., Hoebers N.T., Coumans W.A., Simons P.J., Glatz J.F., Luiken J.J., Larsen T.S. (2015). Marine omega-3 fatty acids prevent myocardial insulin resistance and metabolic remodeling as induced experimentally by high insulin exposure. Am. J. Physiol. Cell Physiol..

[42] Ayerza R., Coates W. (2005). Ground chia seed and chia oil effects on plasma lipids and fatty acids in the rat. J. Endocrinol..

[43] Li N., Wang J., Gao F., Tian Y., Song R., Zhu S.J. (2010). The role of uncoupling protein 2 in the apoptosis induced by free fatty acid in rat cardiomyocytes. J. Cardiovasc. Pharmacol..

[44] Rousseau D., Helies-Toussaint C., Moreau D., Raederstorff D., Grynberg A. (2003). Dietary *n*-3 PUFAs affect the blood pressure rise and cardiac impairments in a hyperinsulinemia rat model *in vivo*. Am. J. Physiol. Heart Circ. Physiol..

[45] Vuksan V., Whitham D., Sievenpiper J.L., Jenkins A.L., Rogovik A.L., Bazinet R.P., Vidgen E., Hanna A. (2007). Supplementation of conventional therapy with the novel grain Salba (Salvia *hispanica* L.) improves major and emerging cardiovascular risk factors in Type 2 diabetes: Results of a randomized controlled trial. Diabetes Care.

